# Crossmodal Congruency Between Background Music and the Online Store Environment: The Moderating Role of Shopping Goals

**DOI:** 10.3389/fpsyg.2022.883920

**Published:** 2022-05-24

**Authors:** Lieve Doucé, Carmen Adams, Olivia Petit, Anton Nijholt

**Affiliations:** ^1^Department of Marketing and Strategy, Faculty of Business Economics, Hasselt University, Diepenbeek, Belgium; ^2^Innovative Entrepreneurship, University College PXL, Hasselt, Belgium; ^3^Marketing and New Consumption Center of Excellence, Kedge Business School, Marseille, France; ^4^Human Media Interaction, University of Twente, Enschede, Netherlands

**Keywords:** crossmodal correspondences, online store atmospherics, shopping goal, congruency, background music

## Abstract

Despite the robust evidence that congruent background music in the physical store environment positively affects consumer reactions, less is known about its effects in an online context. The present study aims (1) to examine whether congruency via multiple elicited crossmodal correspondences between background music and the online store environment (e.g., perceived lightness, loudness, and coldness of the cue/environment) leads to more positive affective, evaluative, and behavioral consumer reactions and (2) to investigate the moderating role of shopping goals on this crossmodal congruency effect. Previous research showed that low task-relevant atmospheric cues like music can have a negative effect on consumers when they visit a website with a purchase goal in mind. An online experiment was conducted with 239 respondents randomly assigned to a shopping goal (experiential browsing vs. goal-directed searching) and a music condition (no music, crossmodally congruent music, or crossmodally incongruent music). Our results show that crossmodally incongruent background music (vs. no music) leads to more positive consumer reactions for experiential browsers and more negative consumer reactions for goal-directed searchers. Conversely, crossmodally congruent background music (vs. no music) has a positive effect on experiential browsers and no adverse effect on goal-directed searchers. Additionally, the presence of crossmodally congruent background music leads to more positive consumer reactions than the presence of crossmodally incongruent background music, independent of the shopping goal. We extend previous research on multisensory congruency effects by showing the added value of establishing congruency between music and the store environment via multiple elicited crossmodal correspondences in the online environment, countering previously found negative effects of low-task relevant atmospheric cues for goal-directed searchers.

## Introduction

The adoption of the internet as a retail channel has risen significantly in the past years, resulting in e-commerce becoming an established value in modern-day Western societies. In the physical store environment, multiple sensory cues like music, scent, or sampling are increasingly used to create pleasant and immersive customer experiences (Biswas et al., 2017). In contrast, at the moment, most online environments are primarily visual and, to a lesser extent, auditory (Petit et al., 2019). Despite the robust evidence that background music in the physical store environment positively affects affective, evaluative, and behavioral consumer reactions (Krishna, 2012; Roschk et al., 2017; Biswas et al., 2019), few online retailers incorporate background music. Since online stores can be visited 24 h a day, everywhere in the world, background music might disturb consumers or others in their environment (e.g., a waiting room or an office space). Moreover, consumers visit online stores for different reasons (Hoffman and Novak, 1996; Moe, 2003; Watson and Popescu, 2021), and previous research showed that the shopping goal significantly moderates the effect of atmospheric cues on consumer responses (Eroglu et al., 2003; Wang et al., 2011). When a consumer visits an online store with a specific purchasing goal in mind, atmospheric cues that are appealing but not relevant to the task (e.g., background music) might decrease the experienced pleasure and satisfaction because it hinders the consumer from achieving his/her goal efficiently.

Another critical element influencing consumer reactions to background music is the congruency or fit between the music and the store environment [e.g., Roschk et al. (2017)]. Previous research in a physical store showed that background music congruent with other atmospheric elements or the overall store environment improves consumer reactions compared to incongruent background music [e.g., Mattila and Wirtz (2001); Helmefalk and Hultén (2017), and Imschloss and Kuehnl (2017)]. Selecting congruent atmospheric elements is, however, not always evident. Most studies have operationalized congruency in terms of the sharing of one specific characteristic like arousing quality (Mattila and Wirtz, 2001) or genre (Krishna et al., 2010). However, perception is fundamentally multisensorial and holistic (Spence et al., 2014). Thus, incorporating multiple sensory characteristics when choosing the appropriate atmospheric cues might enhance consumer reactions. Recently, Adams and Doucé (2017) determined a crossmodal overlap between ambient scent and a physical store environment and found that a pleasant, fitting, and crossmodally congruent scent increased approach behavior compared to a pleasant, fitting, and crossmodally incongruent scent or no scent. However, the question remains whether or not this crossmodal congruency is applicable for other atmospheric cues and in an online store environment.

The present study aims to examine whether crossmodal congruency between background music and the online store environment leads to more positive affective (i.e., pleasure and arousal), evaluative (i.e., online store evaluation), and behavioral (i.e., approach behavior and money spent) consumer reactions, considering the moderating role of shopping goals. We contribute to the existing sensory marketing literature in several ways. First, while the positive effect of congruent background music is well-known for the physical setting [e.g., Krishna (2012), Roschk et al. (2017), and Biswas et al. (2019)], the possible transfer of this knowledge to the online setting needed to be investigated. Second, to our knowledge, congruency between music and the store environment has never been established making use of multiple elicited crossmodal correspondences (e.g., perceived angularity, softness, lightness), which might improve consumer reactions (Spence, 2002). Third, by making use of multiple cue characteristics to create congruency, we argue that adding crossmodally congruent background music to an online store environment does not have a negative effect on goal-directed consumers because crossmodally congruent cues facilitate multisensory integration, leading to improved visual attention and search which helps them achieve their shopping goal efficiently.

The remainder of this paper is organized as follows. First, we will elaborate on relevant research exploring background music effects, crossmodal correspondences, and the moderating role of shopping goals. Next, the materials and methods are presented. Finally, the results, the theoretical and managerial implications, the limitations, and future research directions are discussed.

## Theoretical Background

### Background Music

Nowadays, background music is an indispensable part of the physical store environment. Previous research showed that music in the physical store is indeed a powerful environmental stimulus, triggering positive emotional, cognitive, and behavioral reactions [for an overview, see Garlin and Owen (2006), Roschk et al. (2017), and Biswas et al. (2019)]. To understand these effects of music present in the physical store, various aspects of music have been studied, including tempo (Oakes, 2003; Knoeferle et al., 2012), volume (Kellaris et al., 1996; Guéguen et al., 2008), familiarity (Yalch and Spangenberg, 2000; Bailey and Areni, 2006), liking (Dubé et al., 1995; Broekemier et al., 2008), pitch (Lowe et al., 2019), and genre (Grewal et al., 2003; North et al., 2016). With respect to the use and effects of background music in the online environment, however, research is more limited. In his meta-analysis of atmospheric effects of music, Roschk et al. (2017) analyzed pleasure, satisfaction, and behavioral intention effects of music in 27 retail and service settings compared with only seven online settings. The research done so far indicates, for example, that, in line with the research done in a physical store, the tempo of the background music (Cheng et al., 2009; Ding and Lin, 2012; Anwar et al., 2020) and the genre of the music (Dikèius et al., 2019) are also important elements to consider when choosing the background music for an online store environment.

However, background music is only one element of a larger (online) store environment, and it is important that all atmospheric elements are matched or congruent. Congruency is the fit of a stimulus (i.e., background music) to either a part of or an entire store environment (Roschk et al., 2017). In a physical setting, for example, background music and ambient scent were matched with each other in terms of their arousing quality [i.e., high/high or low/low; Mattila and Wirtz (2001)] or connotation with a holiday [i.e., Christmas music/Christmas scent; Spangenberg et al. (2005)]. This match between background music and ambient scent increased consumers’ experienced pleasure, evaluation of the store, and approach behavior compared to mismatched scent and music conditions. Combinations of music with other cues in the physical store (e.g., the flooring used) also underline the importance of congruency. Imschloss and Kuehnl (2017), for example, found that soft music combined with soft flooring will enhance product evaluations compared to incongruent combinations (e.g., soft music and hard flooring). Additionally, Vida et al. (2007) found that the perceived fit between background music and store image (including the overall atmosphere in the store) increased the length of the shopping time, leading to larger consumers’ expenditure. A positive effect of background music congruent with the coziness and homely atmosphere of the store on pleasure and time spent was also demonstrated by Helmefalk and Hultén (2017) in a physical furnishing store. Overall, prior research provides consistent evidence that in a physical store environment, congruency between two atmospheric elements or between one element and the overall store atmosphere enhances consumer reactions toward the store and its merchandise. In an online store environment, research examining multisensory congruency effects between background music and other atmospheric cues is rather limited. Still, this limited research also signals a favorable effect of congruency. For example, Cheng et al. (2009) found that high arousal music (fast tempo) and color (warm) also led to more pleasure compared to mismatched music and color stimuli, as well as the combination of low arousal music and color (i.e., congruent cues).

Congruency effects can be explained by the fact that music can function as a prime (Oakes, 2007; North et al., 2016). Priming refers to incidental perceptual stimulation that improves the accessibility of concepts that will be used for subsequent information processing (Smeets and Dijksterhuis, 2014). On the one hand, pleasant music can be an affective prime, which means that the music can trigger an overall positive consumer reaction. On the other hand, background music can also function as a cognitive prime. When consumers hear music, an automatic knowledge activation process begins. The music activates related concepts, making them temporarily more accessible and increasing the chance that they will be used in evaluations taken place in the near future (Jo and Berkowitz, 1994; Domke et al., 1998). Previous research showed that music as a focal attribute in advertising (Oakes, 2007) and as a background stimulus in a store environment (North et al., 2016) can semantically prime related concepts. For example, North et al. (2016) found that consumers are more likely to choose and recall a product of a given national origin in the presence of music associated with that specific nation compared to a situation in which music of another national origin is present. Along similar lines, Knoeferle et al. (2016) found that congruent auditory cues such as usage sounds or jingles also facilitate the search and selection of related products in an online store environment. So, music influences consumer behavior by priming conceptually relevant concepts in memory. Cognitive priming might also lead to conceptual fluency when the information activated by the prime (e.g., music) fits with the target element (e.g., store atmosphere). Conceptual fluency is a particular form of processing fluency, indicating the experienced ease of processing an external stimulus (Schwarz, 2004). In particular, it refers to how readily the stimulus comes to mind and how easily its meaning is grasped (Lee and Labroo, 2004). When atmospheric cues are congruent, people can easily process the environment and, as a result, they experience a positive affective state that can be accredited incorrectly to the stimulus rather than to the ease of processing (Winkielman et al., 2003). In contrast, incongruent cues can lead to processing disfluency because the associations activated by the cues do not match each other (Mitchell et al., 1995).

Congruency can thus be achieved in different ways. As mentioned above, music has been matched with other cues based on one cue characteristic, such as their arousing quality [with color: Cheng et al. (2009); with scent: Mattila and Wirtz (2001)] or their association with a holiday [with scent: Spangenberg et al. (2005)]. When music is matched with the overall store atmosphere, the match is generally determined by a global evaluation of the fit between the music and the atmosphere (Demoulin, 2011; Helmefalk and Hultén, 2017). To be able to achieve congruency between an auditory cue and the entire store environment, congruency in terms of the crossmodal correspondences elicited by both may be a possible avenue.

### Crossmodal Correspondences

A crossmodal correspondence is the tendency of one sensory modality to be matched with another sensory modality (Spence, 2012). This means that a cue can be picked up by one sense and trigger an expectation in the same or another sense. For example, Spence (2012) indicates the relationship between high (low) pitch sounds and small (large) size. Hearing a high pitch sound could thus create an expectation of a small object, although the object has not been seen yet. Adams (2018) studied congruency between an atmospheric cue and a retail environment on a crossmodal level. Crossmodal congruency refers to the situation where two different sensory cues evoke the same crossmodal correspondence(s). For example, peppermint scent and the color blue evoke the same crossmodal correspondence of coolness [e.g., Spector and Maurer (2012) and Madzharov et al. (2015)]. Based on a crossmodal congruency index that measures which correspondences (e.g., light vs. dark, loud vs. quiet, cold vs. hot) are elicited by an environmental cue or a whole environment, a crossmodal overlap between an atmospheric cue and a store environment can be determined (Adams and Doucé, 2017; Adams, 2018). In this way, congruency can be established via multiple cue characteristics. Preliminary evidence for the positive effects of crossmodal congruency has been found in a physical store setting in which scent was matched with the environment via the crossmodal congruency index. Adams and Doucé (2017) demonstrated that an ambient scent that elicited the same crossmodal correspondences as the store environment (i.e., crossmodally congruent) had a positive effect on approach behavior compared to a crossmodally incongruent scent. Given the rising importance of online shopping, the current study will investigate whether or not crossmodal congruency between background music and the online store environment leads to more positive consumer reactions. Since congruent atmospheric cues are likely to enhance the ease with which consumers process the environment (Lee and Labroo, 2004; Schwarz, 2004), we offer the following hypotheses:

H1: The presence of crossmodally congruent background music will lead to more positive affective, evaluative, and behavioral consumer reactions than the presence of crossmodally incongruent background music (a) or the absence of background music (b).

With respect to the possible effect of adding incongruent background music, we expect this to be the opposite depending on the type of shopper (experiential browsers versus goal-directed searchers). The hypothesis related to the incongruent background music condition versus the condition of the absence of background music will therefore be presented in the following section after having reviewed the literature concerning the moderating role of shopping goals.

### Moderating Role of Shopping Goal

Shopping goals might affect the relationship between background music and online consumer behavior. Previous research identified different reasons for consumers to visit (online) stores (Hirschman and Holbrook, 1982; Babin et al., 1994; Hoffman and Novak, 1996; Moe, 2003; Rydell and Kucera, 2021; Watson and Popescu, 2021).^1^ Moe (2003) developed and tested a typology of shopping strategies based on two dimensions: search behavior [i.e., goal-directed versus exploratory/experiential behavior; Hoffman and Novak (1996); Janiszewski (1998), and Shih and Jin (2011)] and purchasing horizon (i.e., immediate vs. future). With respect to search behavior, goal-directed behavior (searching) means that the consumer has a specific or planned goal (e.g., purchase) in mind and is motivated to undertake action to achieve his/her goal efficiently. The consumer is very focused and goes through a deliberate process to obtain relevant information. The purchase decision can be immediate or in the future, depending on the amount of information needed to make the decision (related to product involvement). On the other hand, exploratory or experiential behavior (browsing) means that the consumer is less deliberate and is not necessarily considering a purchase. The search is undirected and more stimulus-driven (Janiszewski, 1998), meaning that although the search is not motivated by a specific purchase in mind, the right stimuli might trigger impulse buying. Goal-directed searching is more driven by utilitarian motives related to necessity, rationality, and task completion. Utilitarian shopping value is gained when a consumer buys a product deliberately and efficiently (Hirschman and Holbrook, 1982; Babin et al., 1994). On the other hand, experiential browsing is more driven by the hedonic utility derived from the in-store experience, which is related to fun, playfulness, fantasy, and enjoyment (Hirschman and Holbrook, 1982; Babin et al., 1994; Hoffman and Novak, 1996; Moe, 2003; Shih and Jin, 2011). Hedonic shopping value lies in the shopping experience rather than in the acquisition of goods. With respect to online shopping, the subdivision of hedonic and utilitarian shoppers remains relevant. Depending on the shopping goal, the customer journey of the online customer may include different or additional steps [e.g., third party reviews are more used by the utilitarian shopper, according to Li et al. (2020)] or may induce different purchasing behavior [e.g., impulse buying is more related to hedonic shopping behavior according to Rajan (2020)].

Besides the general effect of shopping goals on behavior, previous research seems to imply that the actual effect of adding music to the online shopping environment may also be dependent on the shopping goal. Eroglu et al. (2003), for example, showed that low task-relevant atmospheric cues (e.g., color, music, entertainment) only led to more pleasure when consumers visit a website for exploratory purposes (i.e., labeled as low involvement) and not when they have a purchasing goal in mind (labeled as high involvement). This finding is in line with the appraisal theory of emotions (Lazarus, 1991), which states that positive emotions can result from the match between environmental stimuli and the individual’s goal. Appealing atmospherics that are not relevant to the purchasing goal can create an unpleasant feeling for goal-directed consumers because it requests more effort to achieve the goal, while it creates more pleasure for experiential browsers because the appealing atmospherics heighten their shopping experience. This was confirmed by Wang et al. (2011), who found that the perceived aesthetic appeal of a website (i.e., the hedonic, attractive, and recreational attributes of the visual design) decreased (vs. increased) satisfaction when a purchase task was pursued (vs. not pursued). However, we argue that background music that is congruent with the store environment based on different elicited crossmodal correspondences (e.g., perceived angularity, softness, and coldness) does not create a distraction or an unpleasant feeling for goal-directed consumers because crossmodal correspondences facilitate multisensory integration (Chen and Spence, 2018; Petit et al., 2019), and therefore improve visual attention and search (Spence, 2011). Thus, visiting an online store with a specific purchase goal makes the consumer focus on the products and the relevant product information. Elements that distract them from achieving their goal will lead to less positive consumer reactions.

Based on the conceptual fluency theory (Lee and Labroo, 2004; Schwarz, 2004), it could be stated that crossmodally incongruent background music is a distraction that creates processing disfluency and cognitive interference and leads to less positive affective, evaluative, and behavioral consumer reactions compared to no music. We do not expect this negative effect to occur in the presence of crossmodally congruent background music because the higher the extent of the fit between sensory stimuli, the more multisensory integration occurs and the easier the store environment is processed (Spence, 2011; Chen and Spence, 2018; Petit et al., 2019). However, we only expect a cancellation of the negative effect and not a positive effect of crossmodally congruent background music compared to no music for goal-directed searchers. The rationale behind this expectation is that goal-directed searchers, as opposed to experiential browsers, are not specifically attracted to pleasant store environments, and the environmental stimuli are still no match with the individual’s goal [cf. appraisal theory of emotions, Lazarus (1991)].

Additionally, consumers who specifically visit an online store for fun, pleasure, and inspiration will also appreciate the presence of pleasant crossmodally incongruent background music because it creates a more pleasant environment that produces sensorial and emotional experiences (Babin et al., 1994; Eroglu et al., 2003). In other words, consumers with a high hedonic shopping motivation desire increased levels of sensory stimulation, causing them to be more attracted to pleasant store environments (Wagner and Rudolph, 2010). We, therefore, posit positive effects for both crossmodally incongruent and congruent background music for experiential shoppers because both music conditions are pleasant and fit with the brand. For goal-directed searchers, on the other hand, we expect the incongruent background music to be a distractor compared to the situation of no music and consequently expect a negative effect.

In sum, we propose that shopping goal moderates the effect of crossmodally (in)congruent background music on consumer reactions toward an online store. More specifically,

H2a: For experiential browsers, the presence of crossmodally incongruent background music will lead to more positive affective, evaluative, and behavioral consumer reactions than the absence of background music, but for goal-directed searchers, it will lead to more negative reactions.

H2b: Only for experiential browsers and not for goal-directed searchers, the presence of crossmodally congruent background music will lead to more positive affective, evaluative, and behavioral consumer reactions than the absence of background music.

The key contributions of the present study to sensory marketing literature are (1) confirming the added value of congruent background music in an online store environment, (2) establishing a more holistic way of determining congruency between background music and the online store environment via various elicited crossmodal correspondences, and (3) showing that crossmodally congruent music cancels the negative effect of background music in an online store setting for goal-directed consumers.

## Materials and Methods

### Research Design and Music Selection

The moderating role of shopping goals on the effect of crossmodally congruent background music on online consumer behavior was examined by a 3 (no music, crossmodally incongruent music, and crossmodally congruent music) × 2 (goal-directed searchers vs. experiential browsers) between-subjects design. As online store environment, an online fashion store targeting men, women, and children was chosen (i.e., H&M). The brand is part of one of the world’s largest fashion retailers, selling online in 53 markets. In the country where the study was conducted, the fashion brand also has a full coverage of physical stores. The online store did not yet make use of music.

A pretest was conducted to find the crossmodally incongruent and congruent musical pieces. A total of 34 respondents (*M*_*age*_ = 25.26; 14 male and 20 female) belonging to the target audience of the brand were recruited via social media (i.e., Facebook). The crossmodal profile of the online store environment and a selection of 10 instrumental pop songs with an equal number of average beats per minute (i.e., approximately 120 bpm, presented in randomized order) were measured by means of the crossmodal congruency index (Adams and Doucé, 2017). The index consists of 11 bi-polar concepts which refer to a certain crossmodal correspondence (i.e., expectation in another sense) that might be triggered by a stimulus (e.g., a store environment, music). The 11 bi-polar concepts represent sensory attributes in the visual sense, the auditory sense, and the tactile sense by means of two antonyms (e.g., loud versus quiet, see Table 1). One of the bi-polar concepts was not exemplified by two words but by the use of a visual representation of a rounded shape (i.e., a spot) versus an angular shape (i.e., a star) in order to determine the elicited crossmodal correspondence of shape. Participants were asked to browse the online shop for at least 2 min and rate the store environment on the elicitation of the 11 bi-polar concepts presented on a 100-millimeter visual analogue scale (VAS). Next, they had to listen to the musical pieces for at least 30 s and rate them on the 11 bi-polar concepts (100-mm VAS) and on a 7-point Likert scale concerning their perceived pleasantness (1 = unpleasant; 7 = pleasant) and their perceived fit with the fashion brand (1 = unfitting; 7 = fitting).

**TABLE 1:** Items of crossmodal congruency index.

First word (left side of VAS)	Second word (right side of VAS)
Star-shape	Spot-shape
Bright	Dim
Cold	Hot
Fragile	Sturdy
High	Low
Light	Dark
Light	Heavy
Loud	Quiet
Rough	Smooth
Shallow	Deep
Soft	Hard

The musical pieces were selected based on three criteria: they should be (1) equally pleasant, (2) equally fitting, and (3) have significantly different crossmodal congruency scores. The profiles of the 10 musical pieces were first analyzed in order to identify those musical pieces which are considered to be pleasant and fitting. The crossmodal profile of the remaining musical pieces was subsequently compared to the crossmodal profile of the online store environment. In line with the calculation method of Adams and Doucé (2017), a crossmodal congruency score was calculated, which represents the degree of congruency between the musical piece and the online store environment. This score was calculated by summing up the absolute difference between the rating of the online store environment and the rating of the musical piece for each of the 11 bi-polar concepts and dividing this sum by 11. The resulting score (between zero and 100) represents the average magnitude of the absolute difference in rating, and consequently, the lower the score, the more crossmodally congruent the musical piece and the online store environment are.

Paired-samples *t*-testing revealed that the instrumental version of *Dancing in the Moonlight* of *Toploader (DM)* and *Get lucky* of *Daft Punk* (*GL)* met these criteria. Both pieces were perceived as equally pleasant [*M*_*DM*_ = 4.76, *SD*_*DM*_ = 1.78; *M*_*GL*_ = 5.21, *SD*_*GL*_ = 1.67; *t*_(33)_ = -1.19, *p* = 0.24] as well as equally fitting with the fashion brand [*M*_*DM*_ = 4.44, *SD*_*DM*_ = 1.71; *M*_*GL*_ = 4.71, *SD*_*GL*_ = 1.90; *t*_(33)_ = -0.70, *p* = 0.49], however, they differed in elicited crossmodal correspondences [*M_*DM*_* = 19.20, *SD*_*DM*_ = 7.43; *M*_*GL*_ = 24.31, *SD*_*GL*_ = 8.65; *t*_(33)_ = -3.70, *p* < 0.001]. *Dancing in the Moonlight* was thus selected as the crossmodally congruent musical piece and *Get lucky* was selected as the crossmodally incongruent musical piece.

### Participants, Procedure, and Dependent Variables

A power analysis for a 3 × 2 ANOVA (medium effect size of 0.25, α = 0.05, power of 0.80, and six groups) showed that the required sample size was 211 participants or 35 participants per condition. A total of 243 respondents were recruited through social media (i.e., Facebook pages of the research team members). They received an invitation to go to the online store at a moment of their choosing, but they were instructed that they should be able to turn their audio on. In exchange for participation, respondents could enter a lottery to win a 25 euros voucher from H&M. Four respondents were deleted because they answered wrong on an attention check question (i.e., a question asking them to answer “slightly agreed”). The final sample consisted of 239 respondents, of which 96% belong to the brand’s target age group of 18–35 years old (*M*_*age*_ = 25.37; 74 male and 165 female). Respondents were randomly assigned to a shopping goal (experiential browsing vs. goal-directed searching) and a music condition (no music, crossmodally congruent music, or crossmodally incongruent music). People in the experiential browser condition had to surf the online store for some inspiration. The respondents were instructed that they could add products to the shopping cart if they saw something they would like to buy. People in the goal-directed searcher condition had to compose an outfit for a night on the town with friends with a maximum budget of 200 euros. While surfing the online store, the respondents heard either the crossmodally congruent music, the crossmodally incongruent music, or no music. There was no time constraint. After completing the shopping task, the participants were asked to fill in an online questionnaire, measuring five dependent variables (i.e., pleasure, arousal, online store evaluation, approach behavior, and money spent).

First, by means of 7-point Likert scales, pleasure and arousal were measured by the items as defined in the Pleasure Arousal Dominance Scale of Mehrabian and Russell (1974). In particular, pleasure was measured by five items (e.g., happy/unhappy; summated scale: α = 0.92) and arousal by three items (e.g., calm/excited; summated scale: α = 0.85). Next, the customers’ overall assessment of the online store was measured by five items on 7-point Likert scales [e.g., bad/good; summated scale; α = 0.95, Spangenberg et al. (1996)]. The five items were adapted from the study of Spangenberg et al. (1996). Approach behavior was measured by eight statements on 7-point Likert scales (summated scale; α = 0.95), based on the study of Donovan and Rossiter (1982), and adapted to an online store environment. Finally, the intended value of money to be spent (i.e., the total value of the shopping cart after completion of the shopping task) was registered. An overview of the scale items and the results of the exploratory factor analysis and reliability analyses are included in the Appendix (Supplementary Material).

## Results

### Influence of Crossmodal Congruency Between Background Music and Online Store Environment

Hypothesis 1 was tested via a MANOVA with music condition as fixed factors and pleasure, arousal, online store evaluation, approach behavior, and money spent as dependent variables (making use of IBM SPSS Statistics). The multivariate test showed a significant main effect of music [Wilks’ lambda = 0.82, *F*_(10,464)_ = 5.91, *p* < 0.001, η_*p*_^2^ = 0.10]. All main effect of music were significant [pleasure: *F*_(2,236)_ = 14.16, *p* < 0.001, η*_*p*_^2^* = 0.11; arousal: *F*_(2,236)_ = 8.10, *p* < 0.001, η*_*p*_^2^* = 0.06; evaluations of the online store: *F*_(2,236)_ = 14.29, *p* < 0.001, η*_*p*_^2^* = 0.11; approach behavior: *F*_(2,236)_ = 12.18, *p* < 0.001, η*_*p*_^2^* = 0.09; money spent: *F*_(2,236)_ = 7.25, *p* < 0.001, η*_*p*_^2^* = 0.06; see Table 2] and the effect sizes indicated medium to large effects. In line with H1, the presence of crossmodally congruent background music led to enhanced pleasure, arousal, evaluations of the online store, approach behavior, and money spent compared to the presence of crossmodally incongruent background music (all Bonferroni corrected *p* < 0.05, except for money spent: Tamhane corrected *p* = 0.41) or the absence of background music (all Bonferroni or Tamhane corrected *p* < 0.05). Although the mean scores indicated that the presence of crossmodally incongruent background music led to a decrease in pleasure, arousal, evaluations of the online store, and approach behavior compared to the absence of background music, the effect was only significant for online store evaluation (Bonferroni corrected *p* = 0.02). These results can be explained by our expectation that the effect of crossmodally incongruent music will be negative for goal-directed searchers and positive for experiential browsers, as indicated in H2a, resulting in a total null effect.

**TABLE 2:** Impact of crossmodal (in) congruent music on affective, evaluative, and approach behavior.

Dependent variables	*F*	*p*	*M* (SD)		
			
			No music*^a^* (*N* = 83)	Crossmodally incongruent music*^b^* (*N* = 77)	Crossmodally congruent music*^c^* (*N* = 79)
Pleasure	14.16	<0.001	4.89*^c^* (1.26)	4.52***^c^*** (1.11)	5.48*^a^****^b^*** (1.02)
Arousal	8.10	<0.001	4.29*^c^* (1.42)	4.10***^c^*** (1.10)	4.93*^a^****^b^*** (1.51)
Online store evaluation	14.29	<0.001	5.13*^bc^* (1.21)	4.59*^a^****^c^*** (1.20)	5.61*^a^****^b^*** (1.15)
Approach behavior	12.18	<0.001	4.70*^c^* (1.25)	4.26***^c^*** (1.30)	5.24*^a^****^b^*** (1.19)
Money spent	7.25	<0.001	96.60***^c^*** (54.06)	119.67 (69.77)	135.77***^a^*** (72.94)

*Bonferroni corrected post-hoc tests were conducted, except for money spent (Tamhane–unequal variances). Superscripts indicate the significant difference at p < 0.05 (in italic when p < 0.01 and in bold when p < 0.001) with the mean of the respective column.*

### Moderating Role of Shopping Goal

To test the moderating role of shopping goals, a 3 × 2 MANOVA with music condition and shopping goal as fixed factors and pleasure, arousal, online store evaluation, approach behavior, and money spent as dependent variables was conducted. The multivariate tests showed a significant interaction effect [Wilks’ lambda = 0.74, *F*_(10,458)_ = 7.34, *p* < 0.001, η*_*p*_^2^* = 0.14], a significant main effect of music [Wilks’ lambda = 0.79, *F*_(10,458)_ = 5.91, *p* < 0.001, η*_*p*_^2^* = 0.11], and a significant main effect of shopping goal [Wilks’ lambda = 0.94, *F*_(5,229)_ = 2.97, *p* = 0.01, η*_*p*_^2^* = 0.06]. A summary of the results of the subsequent univariate analyses can be found in Table 3. All interaction effects [medium to large effect sizes; Cohen (1988)], all main effects of music (all medium effect sizes), and one main effects of shopping goal (on approach behavior, small effect size) were significant (all *p* < 0.05).

**TABLE 3:** Summary of 3 × 2 ANOVA results.

Dependent variables	Model		Music			Shopping goal			Music × Shopping interaction		
				
	*F*_(5,233)_	*p*	*F*_(2,233)_	*p*	η *_*p*_^2^*	*F*_(1,233)_	*p*	η *_*p*_^2^*	*F*_(2,233)_	*p*	η *_*p*_^2^*
Pleasure	15.28	<0.001	16.62	<0.001	0.13	3.55	0.06	0.02	20.24	<0.001	15
Arousal	12.33	<0.001	8.57	<0.001	0.07	3.65	0.06	0.02	19.89	<0.001	0.15
Online store evaluation	17.33	<0.001	17.43	<0.001	0.13	3.38	0.07	0.01	24.77	<0.001	0.18
Approach behavior	14.67	<0.001	14.58	<0.001	0.11	6.81	0.01	0.03	19.46	<0.001	0.14
Money spent	6.38	<0.001	7.24	<0.001	0.06	0.83	0.36	0.01	7.75	<0.001	0.06

**FIGURE 1:**
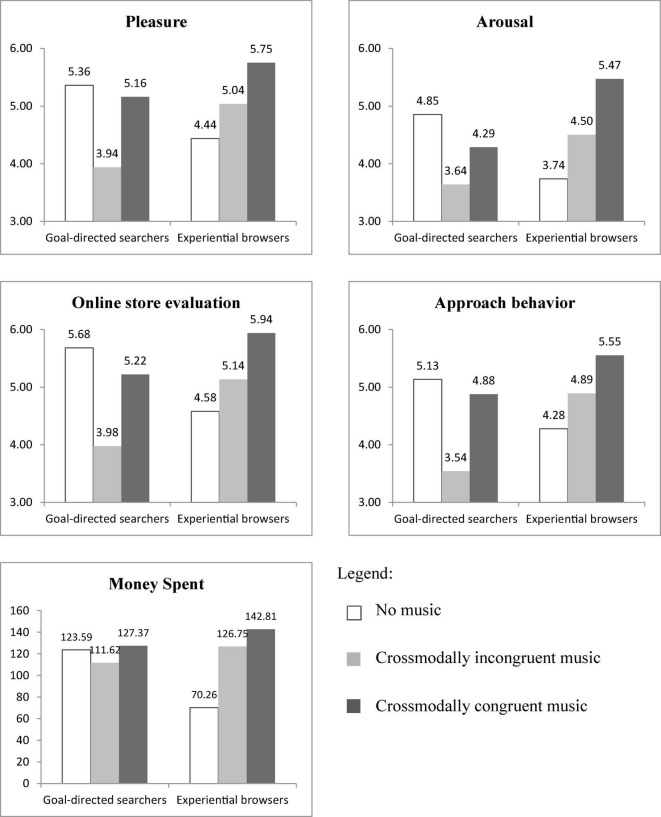
Moderating role of shopping goal on the effect of background music on consumer reactions.

With respect to goal-directed searching, *post-hoc* tests (presented in Table 4 and visualization of means presented in Figure 1) revealed that the presence of crossmodally incongruent background music led to less pleasure (Tamhane corrected *p* < 0.001), less arousal (Tamhane corrected *p* < 0.01), a lower online store evaluation (Tamhane corrected *p* < 0.001), and less approach behavior (Bonferroni corrected *p* < 0.001) than no background music. Goal-directed searchers also spent less money in the crossmodally incongruent music condition, however, these differences were not significant (Tamhane corrected *p* = 0.98). Concerning experiential browsers, the presence of crossmodally incongruent background music increased pleasure (Tamhane corrected *p* = 0.03), arousal (Tamhane corrected *p* < 0.01), and money spent (Tamhane corrected *p* < 0.01) compared to no background music (see Table 4). The differences in online store evaluation and approach behavior were in line with our expectations but did not reach significance (all Bonferroni or Tamhane corrected *p* > 0.14). These results support our expectation that the effect of crossmodally incongruent music is negative for goal-directed searchers and positive for experiential browsers (H2a). There were no differences in the affective, evaluative, or behavioral reactions of goal-directed searchers comparing crossmodally congruent music with no music. For experiential browsers, however, the presence of crossmodally congruent background music had a positive effect on pleasure (Tamhane corrected *p* < 0.001), arousal (Tamhane corrected *p* < 0.001), online store evaluation (Tamhane corrected *p* < 0.001), approach behavior (Bonferroni corrected *p* < 0.001), and money spent (Tamhane corrected *p* < 0.001) compared to no background music, supporting H2b. With respect to crossmodally congruent vs. incongruent music, for both goal-directed searchers and experiential browsers, a positive effect of crossmodally congruent music was expected. Goal-directed searchers experienced more pleasure (Tamhane corrected *p* < 0.001), evaluated the online store more positively (Tamhane corrected *p* < 0.001), and showed more approach behavior (Bonferroni corrected *p* < 0.001) in the presence of crossmodally congruent music compared to crossmodally incongruent music. This positive effect was also found for experiential browsers for pleasure (Tamhane corrected *p* = 0.02), arousal (Tamhane corrected *p* = 0.02), and online store evaluations (Tamhane corrected *p* < 0.01). The means of the other dependent variables were in line with our expectation but did not reach significance (all Bonferroni or Tamhane corrected *p* > 0.15).

**TABLE 4:** Moderating role of shopping goal on the effect of background music on consumer reactions.

	Goal-directed searchers			Experiential browsers		
		
	M (SD)			M (SD)		
		
	No music*^a^* (*N* = 41)	Crossmodally incongruent music*^b^* (*N* = 36)	Crossmodally congruent music*^c^* (*N* = 36)	No music*^d^* (*N* = 42)	Crossmodally incongruent music*^e^* (*N* = 41)	Crossmodally congruent music*^f^* (*N* = 43)
Pleasure	5.36***^b^*** (1.41)	3.94***^ac^*** (1.11)	5.16***^b^*** (0.80)	4.44*^e^****^f^*** (0.88)	5.04*^df^* (0.84)	5.75***^d^****^e^* (1.11)
Arousal	4.85*^b^* (1.72)	3.64*^a^* (1.03)	4.29 (1.16)	3.74*^e^****^f^*** (0.72)	4.50*^d^^f^* (1.02)	5.47***^d^****^e^* (1.56)
Online store evaluation	5.68***^b^*** (1.09)	3.98***^ac^*** (1.29)	5.22***^b^*** (1.02)	4.58***^f^*** (1.09)	5.14*^f^* (0.81)	5.94***^d^****^e^* (1.16)
Approach/Avoidance behavior	5.13***^b^*** (1.20)	3.54***^ac^*** (1.23)	4.88***^b^*** (0.97)	4.28***^f^*** (1.17)	4.89 (1.01)	5.55***^d^*** (1.27)
Money spent	123.59 (49.47)	111.62 (39.45)	127.37 (31.72)	70.26*^e^****^f^*** (44.93)	126.75*^d^* (88.19)	142.81***^d^*** (94.50)

*M, mean; SD, standard deviation. Tamhane post-hoc tests (unequal variances) were conducted, except for approach behavior (equal variances–Bonferroni correction).*

*Comparisons were made between the different music conditions in a specific shopping goal condition. Superscripts indicate the significant difference at p < 0.05 (in italic when p < 0.01 and in bold when p < 0.001) with the mean of the respective column.*

Overall, these results show that pleasant fitting background music increases positive consumer reactions for experiential browsers and that this increase is even higher when the background music is crossmodally congruent with the online store environment. On the other hand, pleasant fitting background music has a negative effect on consumer reactions for goal-directed searchers except if the music is crossmodally congruent with the online store environment.

## Discussion

The aim of this study was (1) to examine whether crossmodal (in)congruency between background music and the online store environment led to more positive (negative) affective, evaluative, and behavioral consumer reactions and (2) to investigate the moderating role of shopping goals on this crossmodal congruency effect. In line with our hypotheses, we found that the presence of crossmodally congruent background music leads to more positive consumer reactions than the presence of crossmodally incongruent background music or the absence of background music. In contrast, the presence of crossmodally incongruent background music only led to more negative evaluations and not to more negative affective and behavioral consumer reactions than the absence of background music. The absence of an overall negative effect of crossmodally incongruent music can be explained by the moderating role of shopping goals. We found that for goal-directed searchers, the presence of pleasant fitting but crossmodally incongruent background music has a negative effect on affective, evaluative, and behavioral reactions (except for money spent). For money spent, the mean scores were in the expected direction, however, the effect did not reach significance. In contrast, for experiential browsers, crossmodally incongruent background music improves affective consumer reactions (i.e., pleasure and arousal) and the amount of money the consumer spent compared to no music. With respect to crossmodally congruent background music (vs. no music), positive effects were found on all measured affective, evaluative, and behavioral consumer reactions for experiential browsers, and no effects (thus also no negative effects) were found for goal-directed searchers. Moreover, crossmodally congruent music leads to more positive affective and evaluative consumer reactions than crossmodally incongruent music for both goal-directed searchers and experiential browsers.

### Theoretical Implications

Our findings confirm that, similar to the physical store environment (Krishna et al., 2010; Roschk et al., 2017), background music in the online store environment can create a pleasant and arousing store environment that consumers evaluate more positively, leading to more approach behavior and spending. However, both crossmodal congruency and shopping goals are important to consider. We extend previous research on multisensory congruency effects by showing the added value of establishing congruency between music and the store environment via multiple elicited crossmodal correspondences (e.g., perceived brightness, deepness, and coldness). Most previous research achieved atmospheric cue congruency via one specific characteristic [e.g., Mattila and Wirtz (2001) and Cheng et al. (2009)], neglecting the multisensorial and holistic nature of perception (Spence et al., 2014). Additionally, our findings extend the positive crossmodally congruent scent effect of Adams and Doucé (2017) to music. With respect to shopping goals, we found that crossmodally congruent background music has no adverse effect on consumers with a specific shopping goal and a positive effect on consumers who browse the store just for fun and inspiration. These results counter previous research stating that low-task relevant atmospheric cues like background music or the aesthetic appeal of a website are not ideal to achieve positive reactions from online consumers with a purchase task (Eroglu et al., 2003; Wang et al., 2011).

### Managerial Implications

Our findings also have practical implications. Few online stores use background music to create a pleasant store environment. One reason for this is that consumers can shop online at every moment and in every location, increasing the chance of disturbing consumers with music that starts playing when entering the website. However, this problem is easily solved by allowing visitors to select and turn music on rather than setting the default to either music or no music. Another reason for the absence of background music is that e-retailers do not always know the shopping goal of the visitors, and previous research showed that appealing atmospheric cues that are not relevant for a purchase task decrease pleasure and satisfaction for goal-directed searchers (Eroglu et al., 2003; Wang et al., 2011). However, our findings show the added value of playing crossmodally congruent background music on retail websites, regardless of the consumer’s shopping goal. Additionally, an e-retailer may, in line with the findings of this paper and the findings of Dikèius et al. (2019) on the effect of genre, consider giving his/her visitors the possibility to choose from an assortment of playlists of which the songs are all crossmodal congruent but belong to different genres (e.g., a playlist of crossmodal congruent popular music and a playlist of crossmodal congruent classical music). In conclusion, for e-retailers, it is important to select the right music considering the crossmodal profile of the music and the online store environment. Heuristic gut feelings and established tools shared among companies that are often used when modifying a store environment (Gigerenzer and Gaissmaier, 2011) should be ignored.

### Limitations and Future Research

A limitation of this study is that the effect of background music was examined in the respondents’ own environment and not in a lab setting. Respondents received an invitation to go to the online store at a moment of their choosing, and they were instructed that they should be able to turn their audio on. Nevertheless, it cannot be guaranteed that all respondents have been exposed to the music during their whole visit. In our design, higher external validity was traded off for less experimental control. Furthermore, the respondents were randomly assigned to a shopping goal (i.e., goal-directed searching: to buy an outfit for a specific occasion or experiential browsing: to surf the online store for some inspiration) and a music condition. Although respondents in the experiential browser condition did not receive a specific goal to buy an outfit, it might be possible that these respondents did have a shopping goal unrelated to the experiment (e.g., to buy outfits they really need). However, because of our research design, these respondents are randomly divided between the three music conditions. Moreover, we did not measure whether or not the respondents used a mobile device and thus interacted with touch screens during their shopping task. Touch screens are sensory interfaces that facilitate haptic interactions and influence search behavior and product choice (Brasel and Gips, 2015; Shen et al., 2016; Chung et al., 2018; Wang et al., 2020). Future research might reveal additional results by considering mobile device use. Another limitation is that we did not investigate the theoretical framework explaining crossmodal congruency effects and the moderating role of shopping motivation. Based on previous research showing that congruent music can prime semantic networks in memory and therefore influence product perception and choice (North et al., 2016) and the finding that the higher the extent of the fit between sensory stimuli, the more multisensory integration occurs, leading to improved visual attention (Spence, 2011), we argued that shopping motivation moderates the effect of crossmodal congruency. However, the underlying processes were not directly tested, and more research is needed to examine these. The present study was the first to use the crossmodal overlap between an atmospheric cue and the overall store environment based on multiple crossmodal correspondences to select background music in an (online) store environment. Future research should investigate whether our results can be replicated using other kinds of music. Moreover, the added value of crossmodal congruency via multiple shared crossmodal correspondences in other retail settings and with other atmospheric cues (e.g., visual cues) should be a future research topic. Finally, it would be interesting to examine other potential moderators (e.g., cognitive load and optimal stimulation level) to understand specific boundary conditions.

## Data Availability Statement

The raw data supporting the conclusions of this article will be made available by the authors, without undue reservation.

## Ethics Statement

Ethical review and approval was not required for the study on human participants in accordance with the local legislation and institutional requirements. The patients/participants provided their written informed consent to participate in this study.

## Author Contributions

LD and CA conceived, planned, and designed the experiment. LD conducted the analyses and drafted the manuscript. CA, OP, and AN provided critical feedback and contributed to the content. All authors approved the submitted version.

## Conflict of Interest

The authors declare that the research was conducted in the absence of any commercial or financial relationships that could be construed as a potential conflict of interest.

## Publisher’s Note

All claims expressed in this article are solely those of the authors and do not necessarily represent those of their affiliated organizations, or those of the publisher, the editors and the reviewers. Any product that may be evaluated in this article, or claim that may be made by its manufacturer, is not guaranteed or endorsed by the publisher.

## Footnotes

1 Moe (2003) uses the term exploratory behavior, whereas Hoffman and Novak (1996) use the term experiential behavior for behavior that is characterized by ritualized orientation, hedonic benefits, non-directed search, and navigational choice.
