# Supplementary material for: Crossmodal Congruency Between Background Music and the Online Store Environment: The Moderating Role of Shopping Goals
Source: Front Psychol. 2022 May 24;13:883920. doi: 10.3389/fpsyg.2022.883920 (PMC9171074; doi:10.3389/fpsyg.2022.883920)
Supplement: Supplementary file 1 [file Table_1.DOCX]

**Appendix**

**Table A1.** Scales of dependent measures and results of exploratory factor analysis and reliability analyses.

| Items | | Cronbach’s alpha | Item loadings |
| --- | --- | --- | --- |
| *Pleasure (5 items; Mehrabian & Russell, 1974)* | | .92 |  |
|  | unhappy/happy |  | .68 |
|  | melancholic/contented |  | .59 |
|  | dissatisfied/satisfied |  | .83 |
|  | despairing/hopeful |  | .79 |
|  | bored/relaxed |  | .71 |
|  | annoyed/pleased |  | x |
| *Arousal (6 items; Mehrabian & Russell, 1974)* | | .85 |  |
|  | calm/excited |  | .75 |
|  | relaxed/stimulated |  | .87 |
|  | unaroused/aroused |  | .87 |
|  | sleepy/wide-awake |  | x |
|  | sluggish/frenzied |  | x |
|  | dull/jittery |  | x |
| *Online store evaluation (5 items; based on Spangenberg et al., 1996)* | | .95 |  |
|  | bad/good |  | .63 |
|  | unfavorable/favorable |  | .54 |
|  | negative/positive |  | .58 |
|  | dislike/like |  | .65 |
|  | outdated/modern |  | .71 |
| *Approach/Avoidance behavior (8 items; based on Donovan & Rossiter, 1982)* | | .95 |  |
|  | I enjoyed shopping in this online store. |  | .69 |
|  | I wanted to stay as long as possible in this store. |  | .67 |
|  | I wanted to leave this store as soon as possible. |  | -.68 |
|  | I would like to revisit this online store in the future. |  | .86 |
|  | It is very likely that I will visit this online store in the future. |  | .83 |
|  | I would recommend this online store. |  | .78 |
|  | I spent more time in the store than I originally intended. |  | .71 |
|  | I spent more money than I originally set out to spend*.* |  | .55 |

*Note.* Items with x were deleted based on a factor (total variance explained = 79.95%) and reliability analysis.
